# The social aspect of water resources management in the Lake Chad Basin: A new construct for water resources management

**DOI:** 10.1371/journal.pone.0327893

**Published:** 2025-07-10

**Authors:** Nodjidoumde Mbaigoto, Kamal A. Alsharif, Shawn Landry, Tara F. Deubel

**Affiliations:** 1 School of Geosciences, College of Arts and Sciences, University of South Florida, Tampa, Florida, United States; 2 Department of Anthropology, University of South Florida, Tampa, Florida, United States; Indian Research Academy, INDIA

## Abstract

Effective water resource management in developing countries requires a nuanced understanding of the social factors that influence public engagement. This study investigates the relationship between socioeconomic and demographic variables and pro-environmental behaviors (PEBs) in the context of Integrated Water Resources Management (IWRM) in N’Djamena, Chad. A household survey of 582 participants was conducted using purposive sampling, and data were analyzed through chi-square tests, logistic regression, and univariate analysis. The findings reveal that while wealth status does not significantly influence PEBs, education level and gender are strong predictors. Individuals with at least a high school education were 2.5 times more likely to participate in water management meetings than those with less education. Men were also significantly more likely to attend such meetings than women. These results underscore the importance of incorporating educational initiatives and gender-sensitive strategies into water governance frameworks. The study recommends early environmental education and the involvement of community and religious leaders to foster inclusive and sustainable water management practices in the Lake Chad Basin.

## Introduction

Fragmented water governance has long been considered a significant barrier to water sustainability, resulting in water crisis [1]. To mitigate the crisis, the 2002 World Summit on Sustainable Development (WSSD) in Johannesburg, South Africa, adopted the Integrated Water Resources Management (IWRM) principles as the road map to water sustainability. IWRM calls for the promotion of coordinated development and management of water, land, and related resources in order to maximize the economic and social welfare in an equitable manner without compromising the sustainability of vital ecosystems and the environment. More than eighty percent of developing countries have integrated IWRM principles into their national water policy and have achieved low to medium levels of IWRM implementation [2]. However, despite the high rate of integration of the IWRM principles into nearly all African countries’ national water policies, most of them will not meet the Sustainable Development Goal 6 (SDG 6) of the 2030 Agenda for Development [3]. According to the 2024 UN Water report, the world is unlikely to achieve sustainable water management until at least 2049, which is 25 years from now. By 2030, an estimated 3.3 billion people will likely lack effective governance frameworks to balance competing water demands and cope with increasing pressures, including those from climate change [4]. The Sustainable Development Goals are the blueprint adopted by world leaders in 2015 at a United Nations summit to achieve a better and more sustainable future for all. The SDG 6 seeks to “ensure availability and sustainable management of water and sanitation for all by 2030” (2).

The Lake Chad Basin Commission (LCBC) has implemented the IWRM principles in its water charter. Still, the region is experiencing one of the worst humanitarian crises, with people displacement, malnutrition, drought, and poor access to health care [5]. Lake Chad is a transboundary lake shared by four riparian countries in Sub-Saharan Africa. The lake has nearly lost 90% of its surface area during the last 40 years. The current surface area is approximately 2000 km^2^ compared to 25,000 km^2^ in 1960 [6]. The shrinkage of Lake Chad threatens the lives of forty million people who depend on the lake’s resources to sustain their livelihood [7].

The population’s perception of water management programs plays a significant role in shaping their pro-environmental behaviors (PEBs), which in turn influence their participation in activities that promote sustainable practices by reducing or eliminating negative environmental impacts. Previous research studies have shown that socioeconomic and demographic factors significantly affect this perception [8]. While weak institutional frameworks based on top-down governance and the lack of coordinated and comprehensive water resources management involving all water users have hindered sustainable development in the region, these institutional and governance frameworks are not the sole contributors to the water crisis in developing countries [9].

Socioeconomic and demographic factors of the population predominantly determine the success rate and people’s perception of natural resources management programs [10.11]. Current water strategies are being implemented without consideration of the local socioeconomic and demographic factors of the population. To ensure the efficiency and sustainability of water management programs, it is crucial to investigate the predominant socioeconomic and demographic factors that are likely to promote positive environmental behavior towards IWRM in the region already suffering from the negative impacts of climate change. The literature review suggested that the failure of IWRM to sustain water resources can be attributed to the lack of participation from low-income and less-educated people in implementing programs aimed at sustainable water resources management. To test the hypothesis, a household survey was conducted in the city of N’Djamena, and the collected data were interpolated into SPSS software to identify education level and three different economic classes: Lower, Middle, and Upper. Frequencies and Chi-Square Test of independence were used to assess the prevalence of one variable over another and evaluate whether there is a relationship between socioeconomic-demographic factors and PEBs.

### Pro-environmental behaviors

Pro-environmental behavior is defined as conscious actions performed by an individual to lessen the negative impact of human activities on the environment or enhance the environment’s quality [12]. Environmental scientists have extensively used behavioral theories such as the Theory of Reasoned Action (TRA) [13] and the Theory of Planned Behavior (TPA) [14] to predict human pro-environmental behavior toward resource management based on social factors [15].

**Fig 1:**
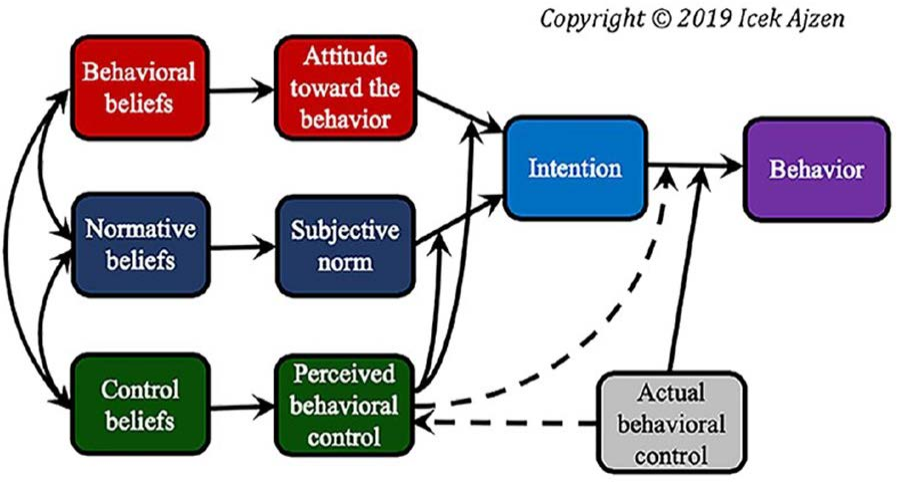
Theory of Planned Behavior (Source Ajzen, 1985).

Both the TRA and the TPB are comprehensive theories that assess the main factors determining human pro-environmental behavior. Those two theories assert that human behavior is guided by rational thought induced by attitudes only when the two refer to the same valued outcome state of being. In other words, attitudes and social norms influence behavioral intentions, which shape people’s actions. The ultimate determinants of any behavior are the behavioral beliefs concerning its consequences and normative beliefs relating to the prescriptions of others [16]. The TRA views behavioral beliefs (attitude towards the behaviors) and normative beliefs (subjective norm) as catalysts that influence people’s intention (ideal construct) to behave in a particular way. The TRA was later developed into the Theory of Planned Behavior (Fig 1), which added control beliefs (perceived behavioral control) as additional catalyzers that control people’s intentions to adopt a pro-environmental behavior.

TPB constructs were found to explain the variance in a workplace between employee intentions to engage in three environmental behaviors [17]. In a similar situation, a study conducted by [18] indicated that Air Force members’ intentions to recycle, conserve energy, and carpool were moderately explained by the tenets of the TPB. Regarding pro-environmental behavior related to participating in water resources management, external constraints and socioeconomic variables were found to be significant moderators of perceived behavioral control when explaining the lack of community participation in government-sponsored programs to conserve riparian resources in Utah [19]. Several studies have pointed out knowledge, education, age, gender, social class, religion, and cultural and ethnic variations as the main factors controlling pro-environmental behaviors [20–24].

### Knowledge and education

Early studies have proven that individuals with more education, in general, are more concerned about the environment and tend to adopt a pro-environmental behavior [25–29]. A study conducted in Chile showed that more educated people tend to be more concerned about the environment and are more willing to adhere to pro-environmental behaviors than those who are less educated [30]. The same conclusion was reached by [31] after a study in Australia found that environmental knowledge positively influences students’ intention to participate in ecotourism activities and landscape likeability. In Ghana, environmental knowledge was found to influence household behavior towards good environmental practices such as green environmental behavior, cleaning environmental behavior, efficient water resource use behavior, and good sanitation behavior [32]. However, other studies have argued that even if possessing or acquiring environmental knowledge can raise people’s concerns, it does not represent a prerequisite to adopting a pro-environmental behavior [12,33,34]. In addition, [12] concluded that knowledge is not correlated with pro-environmental behavior and that “the longer the education, the more extensive is the knowledge about environmental issues. Nevertheless, more education does not necessarily mean increased pro-environmental behavior” [12]. After comparing students with higher environmental knowledge and those with less environmental knowledge, [35] found no significant difference in pro-environmental behavior towards energy saving between the two groups of students.

### Socioeconomic class

In a study [36] suggested that positive environmental behavior correlates with wealth status. The willingness to adopt environmental behavior is higher among affluent people than poor ones. Socioeconomic, demographic, and geographic factors such as age, gender, income, and location were proven to be predominant factors controlling the willingness of the community living near Central Karakoram National Park (Pakistan) to adopt environmental stewardship [37]. Moreover, [38] suggested that economic status plays an essential part in supporting environmental programs. However, it does not represent a prerequisite for support for environmental protection. Wealthy nations are less concerned with financial issues and pay more considerable attention to the preservation of their environment (Park, Beach, etc.). Developing countries, the most vulnerable to pollution, tend to support local environmental programs to improve their livelihood and health. The authors [21] concluded that environmentalism is a global phenomenon independent of a country’s gross national product.

In contrast, a study by [39] found that poor economic groups have a higher perception of susceptibility (an individual’s feeling of being affected by a problem) than wealthy economic groups. The study concluded, “the more susceptible farmers felt about the problem, the more they become aware of the extent of environmental degradation in Haiti, and the more they developed a positive attitude toward solving the problem” [39]. Despite a higher perception of susceptibility among poor people, they are still less likely to change their behavior due to social and economic barriers. Meanwhile, rich people’s behavior toward the environment is subjected to improvement only when they see an immediate and direct threat to their livelihood. Awareness affected all groups, but attitudes towards the problems correlate with high-income categories.

### Gender

Women and men often have differentiated relationships with water access, uses, knowledge, governance, and experiences [40]. Even though a few studies considered gender as a contributing factor in pro-environmental behavior [41,42], most of the studies found that women report stronger pro-environmentalism than men [20,43]. Gender is a significant factor that affects environmental attitude and pro-environmental behavior [12]. These studies unanimously concluded that women tend to behave more pro-environmentally than men [42–46]. This pro-environmental behavior attributed to women is embedded in the nurturing and caregiver characters of women [47]. Women are more likely than men to have internalized altruistic values and dispositions; these altruistic orientations, in turn, guide women to develop higher levels of environmental concern [48]. Conversely, men display a less pro-environmental attitude than women since they are driven by the responsibility of providing economic means to sustain their families [46,47]. However, the predominance of pro-environmental behavior in women cannot be generalized across countries due to the preexistence of internal social factors that play a vital role in women’s behaviors [42,49]. For instance, studies have shown that lower levels of Chinese women’s environmental knowledge relative to men hinder their participation in private environmental behaviors [42].

### Religion

The role of religion in shaping pro-environmental behavior has garnered substantial attention over the past century. Indigenous religions in sub-Saharan Africa, for instance, emphasize water as a communal resource, with rituals and customary laws fostering conservation efforts [50,51]. Similarly, ancestral spiritual beliefs in Thailand have been instrumental in the conservation of natural resources [52]. Islam regards water as a divine gift that must be preserved and shared, with Sharia laws underscoring its moral and communal significance [53]. Despite historical interpretations of dominion over nature, Christianity incorporates a stewardship ethic that advocates for environmental care, viewing water as a symbol of life and purity [54,55].

### The present research: Research aims and hypotheses

This study assessed the impacts of socioeconomic and demographic factors of the Chadian population on adopting pro-environmental behaviors. The population’s socioeconomic and demographic factors are deemed predominant in determining the success rate and people’s perception of natural resources management programs [10,11]. In this study, the term pro-environmental behavior is used interchangeably with the level of participation in any activity that promotes sustainable practices by reducing or eliminating negative environmental impacts. Water governance in Chad is characterized by a top-down approach, with minimal involvement from local communities in the management processes. This governance structure has led to a disconnect between the government and the local populace, which generally perceive the government as solely responsible for water management. However, the adoption of pro-environmental behaviors by the population could significantly enhance water sustainability. To address this gap, various non-profit organizations and local community groups regularly engage with the local population through educational and promotional activities focused on best practices in water management. These events, often conducted as awareness campaigns, aim to educate the community about water-related issues and encourage the adoption of behaviors that support water sustainability. Participation in these meetings allows individuals to gain knowledge that can foster pro-environmental behavior, ultimately contributing to improved water management and sustainability.

This study first categorized the participants into three economic classes, lower, middle, and upper, to assess their impact on PEBs. In Chad, the cultural context prohibits people from asking sensitive questions when conducting surveys. For example, asking direct questions about household income is very personal, and respondents do not usually answer such questions truthfully. Research studies often use proxy indicators of income based on property assets such as televisions, refrigerators, automobiles, and vehicles to evaluate household wealth (economic class). By associating a score index based on the net worth of an asset, it is possible to correlate household assets with economic classes [56]. After the households were classified into three economic classes, the study assessed and determined socioeconomic and demographic factors likely to promote pro-environmental behavior towards IWRM in Chad. This study hypothesizes that low-income and less-educated people in Chad are less likely to participate in the implementation of programs aimed at sustainability and managing water resources.

## Method

### Data collection

A household survey was conducted in N’Djamena, the capital city of Chad. Due to the lack of statistical population data to conduct a random selection (probabilistic methods) in Chad, participants were selected based on a non-probability sampling technique. The Republic of Chad faces significant challenges in maintaining comprehensive demographic data, which hampers researchers’ ability to conduct studies and surveys. Several factors contribute to this situation. Firstly, Chad has not conducted a national census in recent years, making it challenging to apply demographic techniques such as population projection. Secondly, the country’s statistical system suffers from weak institutional frameworks, often based on top-down governance, which limits the capacity to collect, manage, and update demographic data effectively. Additionally, there is a shortage of qualified statisticians and economists in Chad. Lastly, financial limitations restrict the ability to conduct extensive and regular demographic surveys. The costs associated with data collection, processing, and analysis are substantial and often beyond the reach of the national budget. These factors collectively hinder the development and maintenance of reliable demographic data, posing a significant obstacle for researchers aiming to conduct thorough and accurate studies in Chad.

The recruitment period started on 15 August 2019 and ended on 15 September 2019. The interviews and surveys started on September 22, 2019, and continued until October 4, 2019. A non-probability sampling technique selects population samples to be studied based on subjective judgment. For this study, the non-probability sampling technique employed is purposive sampling. Purposive sampling is the preferred method in this study due to the absence of statistical data necessary to randomize the participants’ selection. Purposive sampling enables researchers to deliberately select participants who exhibit specific characteristics or qualities pertinent to the research question. Concentrating on individuals most likely to offer valuable insights ensures that the study can achieve meaningful and reliable results despite the absence of comprehensive statistical data. This approach is particularly advantageous in exploratory research, case studies, or investigations involving unique or hard-to-reach populations, as it allows researchers to gather in-depth information from a targeted group of participants. This method is frequently used in qualitative research to achieve a depth of understanding of social behavior [57]. The study’s goal was clearly explained to the participants, and written consent stating their participation in the survey and the preservation of their anonymity was required before the data collection. The survey consisted primarily of questions related to sociodemographic and economic data such as age, gender, education level, ethnicity, asset ownership, and religion. In addition to demographic data, the survey questionnaire contained open-ended questions intended to measure the population’s participation in water resources management meetings. The University of South Florida Institutional Review Board (IRB) Pro#00039520 reviewed and approved all the survey questions and the research protocol on May 20^th,^ 2019.

A total of 582 households (214 males and 368 females) were selected for this study. Using an effect size (Cohen’s d) of 0.5, an alpha (significance level) of 0.05, and a desired power of 0.80, the calculated power for the sample size (n = 582) is more than sufficient to detect meaningful effects. This ensures high statistical power for the study, enhancing the results’ reliability and validity. The household survey was electronically designed and recorded using Survey123 for ArcGIS. Data captured in Survey 123 for ArcGIS was then downloaded into Statistical Package for the Social Sciences (SPSS) software and the ArcMap platform for further statistical analysis.

**Fig 2:**
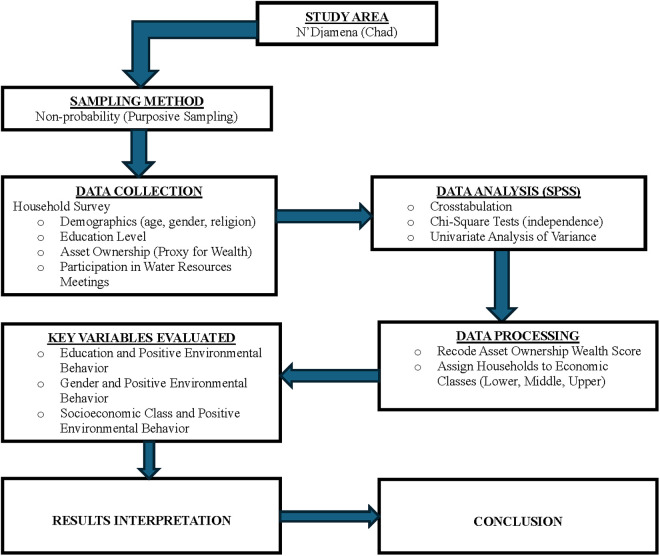
Flow diagram of the study methodology.

The flow diagram (Fig 2) below provides a schematic representation of the methodology used in this study.

### Data analysis

A quantitative analysis was used to assess the correlation between socioeconomic and demographic factors and PEB. Socioeconomic class, education level, and gender were the three factors used in this study to correlate with meeting attendance. Crosstabulation analysis is used to show whether being in one category of the independent variable makes a case more likely to be in a particular category of the dependent variable. Frequencies and Chi-Square (x^2^) Test of independence and Univariate analysis of variance were used to assess the prevalence of one variable over another and evaluate whether there is a relationship (association) between socioeconomic factors and pro-environmental behavior.

## Results

### Socioeconomic class in N’Djamena

Many studies have used property assets as a proxy to estimate household wealth status [58]. In a study [59] used a linear regression model based on household survey data to estimate the income level in Tbilisi, Georgia. The study was first conducted on controlled households with known variables: “income” and “expenditures.” Each owned asset was assigned a score based on its correlation with the two variables. The model was validated and then applied to a larger group in Tbilisi. Results obtained from the study confirmed a linear regression between owned assets scores and income levels. An asset score can be assigned to owned assets based on personal observation, even though this approach is often qualified as apparent objectivity and completely arbitrary [56]. Wealth classification using household assets and subjective perceptions has been widely adopted in large-scale surveys through what is known as the Wealth Index [60]. This method emerged as a practical solution to leverage existing survey data to estimate long-term economic status. It effectively captures disparities in living standards by combining tangible indicators, such as asset ownership and housing quality, with individuals’ own assessments of their financial well-being. Research also highlights that both objective wealth and subjective economic perceptions significantly influence broader social outcomes, including behavior and opportunities [61]. This underscores the importance of integrating both material and perceived dimensions of wealth for a more comprehensive understanding of household economic status. Another approach to assigning a weight or score to owned assets would be using their current value expressed in cash price [56]. However, several factors, such as the purchasing date and price, could affect their current estimated value. Household properties were assessed in this case study to estimate the average income of different economic classes in Chad.

Participants in this study were asked to answer “Yes” or “No” for the ownership of the following assets: Car, House, Electricity, Tap Water, Motorcycle, Bicycle, TV, and Livestock (Table 1). These properties are valuable criteria for estimating household wealth status. Each asset was assigned a unique score based on its monetary value, frequency, and subjective consideration. The scores were assigned to the household assets based on observation and personal experience. Owning a car in Chad is considered prestigious, and only certain classes of society have the privilege to afford to buy a car. Among the surveyed populations, only 11% owned a car. For this reason, a score of “8” is attributed to Car. However, a score of “1” is assigned to the Bicycle as the primary means of transportation for the lower-class society. Owning a Motorcycle is prevalent in N’Djamena and is a common denominator for the middle class. A score of “3” is assigned to the Motorcycle. The score of “7” is given to “House” simply because owning a house in N’Djamena is a sign of wealth. Approximately 65% of the surveyed population owned a home. Buying or building a house in Chad is the first manifestation of a successful life. However, most people usually rent houses instead of buying one due to its high purchasing cost. Electricity is considered a luxury commodity in Chad. Only 8% of the Chadian population has access to electricity. A score of 6 is assigned to electricity. In rural areas, 0.7% of the population has electricity compared to 32% in urban areas. Tap water was given a score of 5. Data collected showed that only 32.3% of the surveyed population have access to the water tap. The households connected to water taps provided by the National Water Company (NWC) are located in urban areas. The limited capacity of the water distribution network through infrastructure renders it impossible for some districts to access the water tap. A score of 4 was assigned to TV because only 26.8% of the surveyed population have a TV. Owning a TV is usually relative to having electricity. Only 6.5 of the survey population owned livestock. Therefore, a score of 2 was assigned to Livestock which mainly consists of cows and goats, and their owners are usually farmers and fishermen living on the city’s outskirts.

**Table 1:** Assets owned.

Asset	Score	Frequency		Percentage (%)	
		Yes	No	Yes	No
**Car**	8	64	518	11	89
**House**	7	376	206	64.6	35.4
**Electricity**	6	251	331	43.1	56.9
**Tap Water**	5	188	394	32.3	67.7
**TV**	4	156	426	26.8	73.2
**Motorcycle**	3	179	403	30.8	69.2
**Livestock**	2	38	544	6.5	93.5
**Bicycle**	1	19	563	3.3	96.7
**Rent**	0	198	384	34	66

“Yes” or “No” answers were recorded for the ownership of household assets. Yes and No values were previously coded in SPSS as “1” for “Yes” and “2” for “No.” Using the transformation tool, the values “1” for each variable were recoded into different variables based on their respective score. The value “2” for not owning the asset was recoded as “0” in the new variable. After recoding the values into different variables, a new variable named “Wealth Score” was created for each household. The value of the “Wealth Score” for each household is nothing but the sum of every owned asset’s scores within that household (Table 2).

**Table 2:** Wealth score distribution among households surveyed.

		n	%	Cum%
**Wealth Score**	0	96	16.5	16.5
	1	2	.3	16.8
	3	19	3.3	20.1
	4	1	.2	20.3
	5	12	2.1	22.3
	6	6	1.0	23.4
	7	154	26.5	49.8
	8	2	.3	50.2
	9	16	2.7	52.9
	10	15	2.6	55.5
	11	12	2.1	57.6
	12	10	1.7	59.3
	13	16	2.7	62.0
	14	3	.5	62.5
	15	19	3.3	65.8
	16	12	2.1	67.9
	17	7	1.2	69.1
	18	32	5.5	74.6
	19	7	1.2	75.8
	20	17	2.9	78.7
	21	14	2.4	81.1
	22	20	3.4	84.5
	23	2	.3	84.9
	24	3	.5	85.4
	25	43	7.4	92.8
	26	8	1.4	94.2
	27	3	.5	94.7
	28	2	.3	95.0
	30	16	2.7	97.8
	31	2	.3	98.1
	32	1	.2	98.3
	33	5	.9	99.1
	35	3	.5	99.7
	36	2	.3	100.0
	**Total**	582	100.0	

(N = 582, M = 11.82, SD = 9.163).

The highest and lowest wealth scores observed were, respectively, 36 and 0. The wealth score mean (*M*) is 11.82 with a St. Deviation (*SD*) of 9.163. Using mean ± standard deviation (SD) to classify wealth status is often preferred when the data follows a normal distribution, as it assumes symmetry around the mean. This method enables a clear categorization of individuals into low wealth (below mean − SD), middle wealth (within mean ± SD), and high wealth (above mean + SD). It aligns with standard statistical practices, enhancing the interpretability and comparability of findings across studies. Additionally, mean ± SD captures the variability and dispersion within the dataset, making it more sensitive to subtle differences in wealth levels than fixed quantile-based methods like tertiles or quartiles, which may overlook important nuances in normally distributed data. Based on the results, three economic classes were classified: upper, middle, and lower classes. The mean value and the standard deviation were used to represent the middle class. Wealth scores comprised within the mean (11.82) and ± Std. Deviation (9.163) is referred to as the middle class (3–21). Values higher than mean + Std. Deviation (> 21) and lower than mean -Std. Deviation (< 3) are respectively categorized as upper and lower class. Each household was then assigned a socioeconomic class based on its wealth score. Socioeconomic classes in the study area are then distributed as follows: 18.9% upper class, 20.10% lower class, and 61% middle class.

### Pro-environmental behavior and socioeconomic class

The distribution of regular meeting attendance across socioeconomic groups is presented in Table 3. It indicates that 17.1% of individuals from the lower class, 22% from the middle class, and 20% from the upper class participate in meetings concerning water and environmental issues within their district. A logistic regression analysis was conducted to examine whether socioeconomic status significantly predicts meeting attendance. The results revealed no statistically significant relationship between economic class and meeting participation, χ² (2, N = 582) = 1.31, p = .051. Furthermore, the model’s explanatory power was minimal, as evidenced by the Cox & Snell R² = 0.002 and Nagelkerke R² = 0.004, suggesting that less than 1% of the variance in attendance is accounted for by economic class. Given that the p-value exceeds the conventional threshold for significance (α = 0.05), the null hypothesis cannot be rejected. Additionally, the confidence interval [0.05, 7.38] encompasses values consistent with no effect, reinforcing the conclusion that socioeconomic class is not a significant predictor of meeting attendance.

**Table 3:** Cross tabulation of socioeconomic class and meeting participation.

			Meeting attendance		Total
			Yes	No	
**Economic Class**	**Lower Class**	n	20	97	117
		%	17.1%	82.9%	100.0%
	**Middle Class**	n	78	277	355
		%	22.0%	78.0%	100.0%
	**Upper Class**	n	22	88	110
		%	20.0%	80.0%	100.0%
**Total**		n	120	462	582
		%	20.6%	79.4%	100.0%

Chi (2) = 1.311, p = 0.519.

Furthermore, Univariate analysis of variance was run to measure the effect size (a measure of association) of the “wealth score” variable on the “meeting participation” variable (Table 4). The value of Eta Squared (η^2 ^= 0.000018) explains that the variance in the participation level is not attributable to wealth score. The η^2^ value is very low to suggest there is an association between wealth score and participation level. Both the Pearson Chi-Square and the Eta Squared showed that wealth is not a defining factor in population participation level in the management of the Lake Chad Basin.

**Table 4:** Effect size between wealth score and meeting attendance.

Dependent Variable: Wealth Score						
Source	Type III Sum of Squares	df	Mean Square	F	Sig.	Partial Eta Squared (η^2^)
**Corrected Model**	.856^a^	1	.856	.010	.920	.000
**Intercept**	53528.774	1	53528.774	636.447	.000	.523
**Attendance**	.856	1	.856	.010	.920	.000
**Error**	48781.268	580	84.106			
**Total**	130160.000	582				
**Corrected Total**	48782.124	581				

^a^R Squared = .000 (Adjusted R Squared = −.002).

### Pro-environmental behavior and education

The age distribution among the studied population varied from 18 to 100, with the participants’ ages predominantly between 26 and 35 (33.5%) and 36 and 50 (30.4%). The survey data showed that 14.4% of participants have never attended school, 9.1% have elementary education, 13.1% attended middle school, 19.4% have a high school degree, and 42.3% attended college (Table 5).

**Table 5:** Household Survey data.

Socio-Demographic Characteristics	n		%		Cum%
***Gender***					
Female	214		36.8		36.8
Male	368		63.2		100.0
N	582		100.0		
***Age***					
18-25	109	18.7		18.7	
26-35	195	33.5		52.2	
36-50	177	30.4		82.6	
51-75	96	16.5		99.1	
76-100	5	0.9		100.0	
N	582	100.0			
***Education***					
Never Attended School	84	14.4		14.4	
Elementary School	53	9.1		23.5	
Middle School	76	13.1		36.6	
HS Graduate	113	19.4		56.0	
Trade School	10	1.7		57.7	
Associate	35	6.0		63.7	
Bachelor	157	27.0		90.7	
Master	49	8.4		99.1	
Doctorate	5	0.9		100.0	
N	582	100.0			
***Religion***					
Animist	5	0.9		0.9	
Christian	347	59.6		60.5	
Muslim	214	36.8		97.3	
Others	16	2.7		100.0	
N	582	100.0			

A Chi-Square test of independence was conducted to determine whether meeting attendance is related to education level (Table 6). The relation between these two variables was significant, *X*^2^(8, *N* = 582) = 24.02, *p* < .05. Education level plays a vital role in meeting attendance among the study population. When exploring individual education levels, participants (40%) with a doctorate are more likely to participate in meetings than those who never attended school (8.3%). However, the results also indicated that participants with high school degrees (23%) are more likely to participate in meetings than those who have, respectively, an associate degree (14.3%), trade school certification (10.0%), or less than a high school degree.

**Table 6:** Crosstabulation of education level and meeting attendance.

			Meeting Attendance^a,^		Total
			Yes	No	
**Education Level**	**Never Attended School**	n	7	77	84
		%	8.3%	91.7%	100.0%
	**Elementary School**	n	7	46	53
		%	13.2%	86.8%	100.0%
	**Middle School**	n	12	64	76
		%	15.8%	84.2%	100.0%
	**HS Graduate**	n	26	87	113
		%	23.0%	77.0%	100.0%
	**Trade School**	n	1	9	10
		%	10.0%	90.0%	100.0%
	**Associate**	n	5	30	35
		%	14.3%	85.7%	100.0%
	**Bachelor**	n	43	114	157
		%	27.4%	72.6%	100.0%
	**Master**	n	17	32	49
		%	34.7%	65.3%	100.0%
	**Doctorate**	n	2	3	5
		%	40.0%	60.0%	100.0%
**Total**		n	120	462	582
		%	20.6%	79.4%	100.0%

^a^Chi (8) = 24.023, p = .002.

Given the non-linear relationship between education level and meeting attendance, the education variable was manually recoded into two categories: individuals with less than a high school diploma and those with a high school diploma or higher (Table 7). A logistic regression analysis revealed a statistically significant association between education level and meeting attendance, χ²(1, N = 582) = 14.524, p < .001. The negative regression coefficient (B = −0.900) indicates that individuals with less than a high school education are significantly less likely to attend meetings. The odds ratio (Exp(B) = 0.407) suggests that participants with at least a high school education are approximately 2.5 times more likely to attend meetings concerning water issues than those with lower educational attainment.

**Table 7:** Education level (Binning)* meeting attendance crosstabulation.

			Meeting Attendance^b^		Total
			Yes	No	
**Education Level** ^**a**^	**Below High School**	n	26	187	213
		%	12.2%	87.8%	100.0%
	**High School and Above**	n	94	275	369
		%	25.5%	74.5%	100.0%
**Total**		n	120	462	582
		%	20.6%	79.4%	100.0%

^a^Education Level Binning from Household Survey Data.

^b^Chi (1) = 14.524, p = .000138.

### Pro-environmental behavior and gender

The results show that 14% of women and 24.5% of men regularly attend water meetings. The regression analysis (X2(1) = 9.00, p = .003) indicates that gender contributes meaningfully to predicting meeting attendance (see Table 8). The negative coefficient for gender (B = −0.686) indicates that females are less likely to attend meetings compared to males. The odds ratio (Exp(B) = 0.504) suggests that females are about 50% as likely as males to attend water and environmental issues meetings. There is a statistically significant correlation between gender and meeting attendance. Specifically, males are significantly more likely to attend these meetings than females.

**Table 8:** Crosstabulation gender and meeting participation.

			Meeting Attendance^b^		Total
			Yes	No	
**Gender**	**Female**	n	30	184	214
		%	14.0%	86.0%	100.0%
	**Male**	n	90	278	368
		%	24.5%	75.5%	100.0%
**Total**		n	120	462	582
		%	20.6%	79.4%	100.0%

^b^Chi (1) = 9.007, p = .003.

### Pro-environmental behavior and religion

The survey data reveals a significant disparity in participation in community meetings among different religious groups. In particular, Muslim women in Chad often faced restrictions that limited their ability to engage with the survey team. Many were not permitted to speak in the presence of their husbands, despite being primarily responsible for managing and supplying domestic water for household use. Some Muslim women agreed to participate only under specific conditions—namely, when their husbands were absent and the interviewer was a woman wearing a hijab.

Among the women who completed the survey, 36% identified as housewives whose husbands were not at home during the data collection period. A logistic regression analysis comparing Christian and Muslim participants regarding meeting attendance yielded a p-value of 0.078. Although this result is marginally non-significant, it suggests a weak or inconclusive relationship between religious affiliation and participation in community meetings.

While religion appears to influence meeting attendance, the data do not provide sufficient statistical evidence to establish a definitive correlation between religious identity and pro-environmental behavior. Although there is a slight trend indicating that Christian participants may be more likely to attend meetings than their Muslim counterparts, the evidence is not robust enough to support a conclusive interpretation.

## Discussion and conclusion

This study examined the influence of key socioeconomic and demographic variables—namely, education, gender, and religion—on pro-environmental behaviors (PEBs) in N’Djamena, Chad, within the framework of Integrated Water Resources Management (IWRM). The analysis revealed that education is a significant predictor of PEBs, with individuals possessing at least a high school education being 2.5 times more likely to participate in water-related community meetings. Gender also emerged as a critical factor, with men significantly more likely than women to engage in such meetings. There is gender inequality in water resources management in the Lake Chad Basin. Men tend to participate more in water resources management than women do. The weight of cultural norms and values in the study area prevented the full participation of all community members in the decision-making process. Men are considered the head of households and are responsible for productive activities outside the home. Meanwhile, women’s tasks are mainly relegated to the house and consist of caregiving and motherhood. Although religion did not exhibit a statistically significant effect, qualitative insights suggest that cultural norms, particularly within Muslim communities, may inhibit women’s participation in environmental governance. Interestingly, wealth status did not significantly influence PEBs, challenging assumptions that economic capacity directly correlates with environmental engagement.

The findings of this study are consistent with a growing body of literature that underscores the role of education in fostering environmental awareness and participatory behavior. Prior research has demonstrated that higher levels of education are associated with increased environmental concern and action, a relationship that aligns with the Theory of Planned Behavior (TPB), which posits that knowledge and perceived behavioral control are key determinants of pro-environmental intentions and behaviors [14,15,25,32]. The observed gender disparity also mirrors global trends, where women often express greater environmental concern but face structural and cultural barriers to participation [43,48]. In the context of Chad, these barriers appear to be compounded by religious and cultural norms that limit women’s public engagement, particularly in Muslim communities [42,49].

Contrary to some studies conducted in more affluent or urbanized settings, this study found no significant relationship between wealth and PEBs [36–38]. This divergence may reflect the unique socioeconomic dynamics of developing regions, where environmental concern is not necessarily contingent on economic affluence. Instead, systemic constraints such as limited access to resources and institutional support may inhibit behavioral change, even among those with environmental awareness [39].

Understanding the relationship between socioeconomic factors, such as education, income, and gender, and pro-environmental behaviors requires careful consideration of confounding variables that may influence or obscure these associations. Cultural norms, religious beliefs, and external economic pressures often interact with socioeconomic status in complex and context-specific ways. In Chad, religion significantly shapes educational access, particularly for girls. Traditional Islamic households may prioritize Qur’anic education over secular schooling, limiting exposure to subjects like science, environmental stewardship, and civic engagement. This educational gap can hinder awareness and participation in environmental initiatives, such as community-based water management. Additionally, entrenched gender norms may restrict women’s involvement in formal environmental decision-making, despite their central role in managing household water resources. Economic hardship further complicates this dynamic, as individuals facing poverty or food insecurity may prioritize immediate survival over long-term environmental concerns, regardless of their educational background. Therefore, while education and gender are important predictors of PEBs, they do not operate in isolation. A nuanced understanding of how religious traditions, cultural expectations, and economic realities intersect with socioeconomic status is essential for designing inclusive and effective environmental policies.

The findings have important implications for water governance in the Lake Chad Basin, a region facing acute environmental and socio-political challenges. First, the results highlight the inadequacy of top-down governance models that fail to account for local sociocultural dynamics. Effective community engagement strategies must be context-specific, recognizing the region’s diverse educational, gendered, and religious landscapes [62]. Religious institutions have a modest but meaningful influence on encouraging pro-environmental behaviors (PEBs). Therefore, it is strongly recommended that mosque and church leaders take time to educate their congregations on sustainable practices, particularly those that support water conservation. A similar initiative in Malaysia showed that involving religious leaders in sustainability efforts can significantly boost public participation in environmental programs [63]. Second, the strong association between education and PEBs underscores the need to integrate environmental education into formal and informal learning systems. Early exposure to environmental issues can foster long-term behavioral change and community stewardship. Third, the gender gap in participation necessitates the adoption of gender-sensitive approaches that empower women as key stakeholders in water governance. Despite their central role in household water management, women remain underrepresented in decision-making processes, a gap that must be addressed through inclusive policy design and implementation.

### Study limitations

Several limitations should be acknowledged. The use of non-probability purposive sampling, while necessary due to logistical constraints, limits the generalizability of the findings. This sampling method may introduce selection bias, as participants were not randomly selected. Additionally, the reliance on self-reported data raises concerns about social desirability bias, particularly in relation to sensitive topics such as religion and gender roles. Respondents may have over- or under-reported their behaviors to align with perceived social norms. Furthermore, it is highly important to consider the impacts of some control variables (religion, governance arrangements, environmental and geographic location of participants, etc.) when assessing their PEBs. Those control variables were proven to [64]. Finally, the cross-sectional nature of the study precludes causal inference. Further longitudinal research is needed to assess how PEBs evolve over time and in response to targeted interventions.

## Supporting information

S1 Copy of data collected final. (XLSX)
